# Phylogenetic typing and molecular detection of virulence factors of avian pathogenic *Escherichia coli* isolated from colibacillosis cases in Japanese quail

**Published:** 2017-03-15

**Authors:** Hesam Alizade, Reza Ghanbarpour, Maziar Jajarami, Asma Askari

**Affiliations:** 1 *Infectious and Tropical Disease Research Center, Hormozgan Health Institute, Hormozgan University of Medical Sciences, Bandar Abbas, Iran; *; 2 *Research Center for Tropical and Infectious Diseases, Kerman University of Medical Sciences, Kerman, Iran; *; 3 *Department of Pathobiology, Faculty of Veterinary Medicine, Shahid Bahonar University, Kerman, Iran; *; 4 *Zoonosis Research Committee, Kerman University of Medical Sciences, Kerman, Iran.*

**Keywords:** Colibacillosis, *Escherichia coli*, Japanese quail, Phylogenetic group, Virulence gene

## Abstract

Colibacillosis caused by avian pathogenic *Escherichia coli* (APEC) is an economic threat to the poultry industry throughout the world. Some of the virulence genes may enhance the ability of *E. coli* isolates to grow in the tissues of broilers. The APEC strains are assigned to a few distinct phylogenetic groups. The purpose of the present study was to detect the virulence genes and phylogenetic groups of *E. coli *isolates from colibacillosis cases in Japanese quail in 2014 in Kerman, Iran. In the present study, one hundred and two *E. coli* isolates were obtained from dead Japanese quails with colibacillosis. *E. coli *isolates were confirmed by standard biochemical and bacteriological methods. DNA of *E. coli *isolates was extracted by boiling method. The confirmed *E. coli* isolates were investigated to detect the phylogenetic groups and virulence genes including *sfa/focDE*, *afaIBC*,* papEF *by PCR methods. *E. coli* isolates were classified into A (62 isolates), B1 (24 isolates), B2 (12 isolates) and D (four isolates) phylogenetic groups. Among examined isolates nine isolates (8.82%) were positive for *papE-F*, five isolates (4.90%) for *afaIB-C* and two isolates (1.96%) for *sfa/focD-E* genes. Based on our findings, *E. coli* isolates from colibacillosis of Japanese quail could be assigned to various phylogenetic groups (mostly A and D), and they may contain the adhesion genes in a low prevalence.

## Introduction

Avian pathogenic *Escherichia coli* (APEC) are responsible for a variety of extra-intestinal pathogens in poultry, including colibacillosis, yolk sac infection, cellulitis, coli-granuloma and omphalitis.^1^ Although Japanese quail (*Coturnix coturnix japonica*) are reported as resistant birds against many diseases, APEC strains have been isolated from colisepticemic poultry with colibacillosis.^1^ Colibacillosis is an economic threat to the poultry industry which is a worldwide infection.^2^ Virulence factors (VFs) of extra-intestinal pathogenic *E. coli *(ExPEC) participate in colonization, cellular invasion and consequently reduction of the host immunity responses.^3^ Some of the VFs such as fimbrial antigens (P, AC/I, F1A and Stg), iron acquisition systems (aerobactin) and toxins (cytolethal distending toxins and hemolysins) may enhance the ability of *E. coli* to grow in the tissues of broilers.^4^ Expression of adhesions such as S and P fimbriae are considered to be an essential factor in pathogenesis of these strains because of their fundamental abilities for the adherence to the epithelium cells of birds.^5^ Stordeur *et al*. reported fimbrial and afimbrial adhesion genes normally expressed in extra-intestinal and intestinal strains isolated from birds.^6^ P fimbriae are an important step for the beginning and expansion of human urinary tract infections, but their role in pathogenesis of avian isolates has not been elucidated, completely.^5^ The *E*. *coli *strains are genetically diverse, and strains have been divided into four major phylogenetic groups (A, B1, B2, and D).^7^ According to the phylogenetic analysis most ExPEC strains are assigned to phylogenetic group B2 and to a lesser extent, to group D and possess a panel of VFs such as adhesins.^8^^,^^9^

The aims of present study were screening of the virulence genes *papE-F*, *sfa/focD-E* and *afaIB-C* and phylogenetic grouping of *E. coli *isolates from colibacillosis cases in Japanese quail in Kerman, Iran.

## Materials and Methods

**Bacterial isolates. **In this cross-sectional study, a total number of 212 dead cases of Japanese quail were collected from the Eslam-Kish farm in Kerman province in 2014. Dead Japanese quail swabs were collected aseptically for the isolation of bacteria. Isolation of *E coli* was done from heart blood, liver, or typical visceral lesions in a fresh carcass. Samples were streaked on MacConkey and eosin-methylene blue (EMB) agar (Biolife Laboratories, Milan, Italy). *Escherichia coli *isolates were confirmed by standard biochemical and bacteriological methods. All of the *E. coli* isolates were stored in Luria-Bertani broth (Invitrogen, Paisley, Scotland) with 25% sterile glycerol (Pars Behbood Asia, Mashhad, Iran) at –70 ˚C.

**Detection of virulence genes. **DNA was extracted from the *E. coli *isolates and reference strains by the boiling method. The *E. coli* isolates were analyzed for the presence of the *papE-F*, *sfa/focD-E* and *afaIB-C* genes by PCR method as described previously.^10^ The reference strains were used as positive controls for virulence genes including 28C (*papE-F*), A30 (*afaIB-C*) and J96 (*sfa/focD-E*). The *E. coli* strain MG1655 was used as a negative control.

**Phylogenetic groups. **The phylogenetic analyses of the isolates were determined by presence and/or absence of the three genetic markers, *chuA*, *yjaA*, and TSPE4.C2 by a triplex PCR as described by Clermont *et al*.^7^ The isolates of *E. coli* were segregated in four distinct phylogenetic groups: A, B1, B2 and D. Four *E. coli* strains from the ECOR collection were used as controls for phylogenetic determination: ECOR58 (B1 group), ECOR62 (B2 group), ECOR50 (D group) and *E. coli* strain MG1655 as a positive control for phylogenetic ECOR group A. The primers used for amplification of the virulence genes and phylogenetic groups are shown in Table 1. The reference strains were from the bacterial culture collection, Department of Microbiology, School of Veterinary, Toulouse, France.

**Table 1:** Oligonucleotide primers used in this study

**Genes**	**Primer Sequence (5** **′** **-3** **′** **)**	**Product size (bp)**
***afaIBC***	GCTGGGCAGCAAACTGATAACTCTC	750 bp
	CATCAAGCTGTTTGTTCGTCCGCCG	
***sfa/focDE***	CTCCGGAGAACTGGGTGCATCTTAC	410 bp
	CGGAGGAGTAATTACAAACCTGGCA	
***papEF***	GCAACAGCAACGCTGGTTGCATCAT	336 bp
	AGAGAGAGCCACTCTTATACGGACA	
***yjaA***	TGAAGTGTCAGGAGACGCTG	211 bp
	ATGGAGAATGCGTTCCTCAAC	
**TspE4C2**	GAGTAATGTCGGGGCATTCA	152 bp
	CGCGCCAACAAAGTATTACG	
***chuA***	GACGAACCAACGGTCAGGAT	279 bp
	TGCCGCCAGTACCAAAGACA	

## Results

**Fig. 1:**
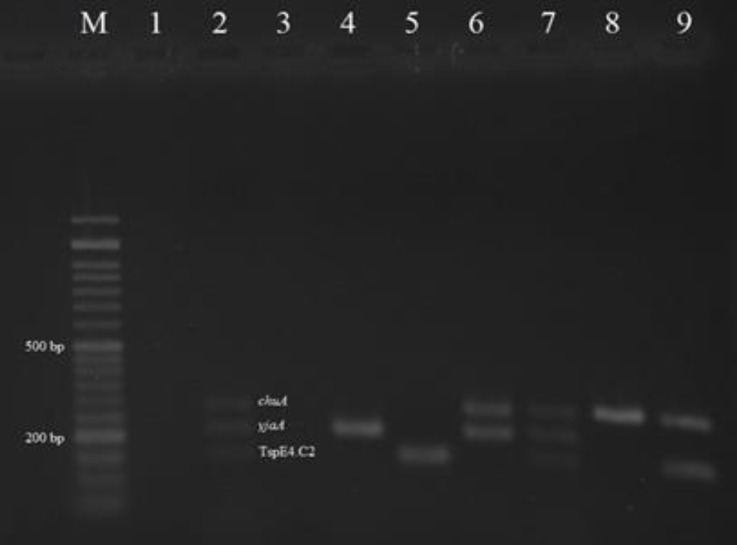
Multiplex PCR results for the detection of *E. coli *phylogenetic groups among the colibacillosis cases of Japanese quail; Lane M: Ladder 50 bp; Lane 1: Negative control *E. coli *MG1655; Lane 2: Positive control *E. coli *ECOR62; Lanes 3, 4: A phylo-group; Lane 5: B1 phylo-group; Lanes 6, 7: B2 phylo-group; Lanes 8, 9: D phylo-group

Among 212 dead cases of Japanese quail, pure colonies of *E. coli* were obtained from 102 samples in MacConkey and EMB agar. The recovered isolates were confirmed as *E. coli* based on standard bacteriological and biochemical tests. PCR analysis indicated that the 102 *E. coli* isolates assigned to the phylo-groups A (62 isolates; 60.78%), B1 (24 isolates; 23.52%), B2 (12 isolates; 11.76%) and D (4 isolates; 3.92%), (Fig.1). 

**Fig. 2:**
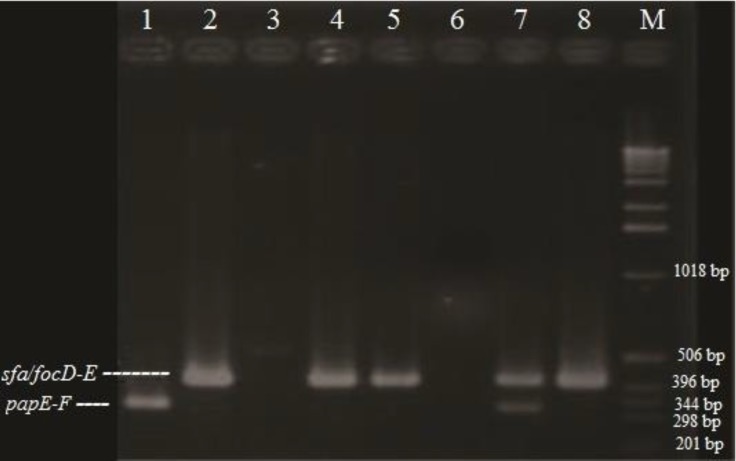
The multiplex PCR results for *sfa/focD-E *and *papE-F *genes; Lane M: ladder 1Kb; Lane 1: Positive control *E. coli *28C; Lane 2: Positive control *E. coli *J96; Lane 3: Negative control *E. coli *MG1655; Lanes 4, 5, 8: Positive isolates for *sfa/focD-E *gene; Lane 7: the Positive isolate for both *sfa/focD-E *and *papE-F *genes

**Fig. 3:**
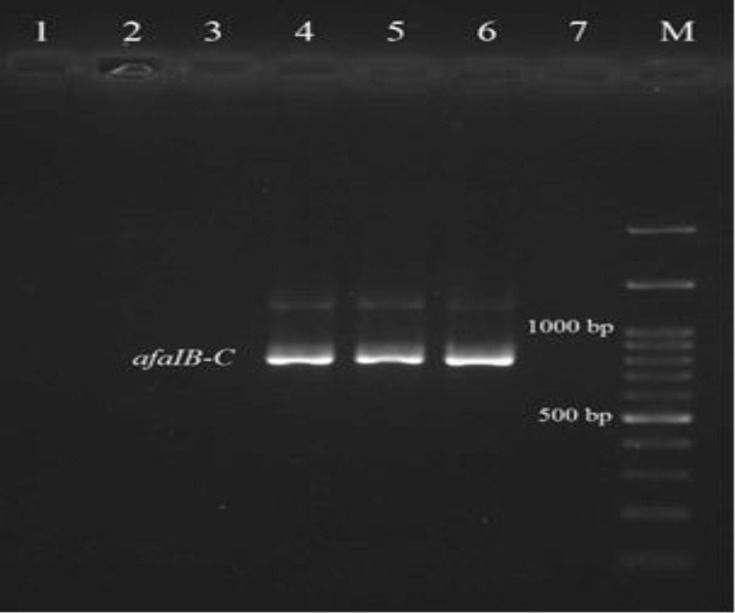
The PCR results for *afaIB-C* gene; Lane M: Ladder 100 bp; Lanes 4, 5: Positive isolates for *afaIB-C* gene; Lane 6: Positive control *E. coli *A30; Lane 7: Negative control *E. coli *MG1655

Virulence genotyping of *E. coli* isolates showed that fourteen of the isolates exhibited at least one of the virulence genes. Multiplex PCR assay revealed that nine isolates (8.82%) were positive for *papE-F*, five isolates (4.90%) for *afaIB-C* and two isolates (1.96%) for *sfa/focD-E* genes (Figs. 2 and 3).

Overall, out of 102 *E. coli* isolates, nine isolates (8.82%) were positive for P fimbriae coding gene. These isolates segregated in phylogenetic groups A (two isolates; 22.22%), B1 (one isolate; 11.11%), B2 (5 isolates; 55.55%) and D (one isolate; 11.11%). *afaIB-C* gene was detected in 4.90% of isolates, which fell into A (40.00%), B1 (20.00%) and B2 (40.00%) phylogenetic groups. Two positive isolates for S fimbriae coding fell into B1 phylogenetic group. Two isolates were positive for both *papE-F *and *sfa/focD-E* genes, which belonged to two phylo-groups including B1 (n = 1) and B2 (n = 2). 

## Discussion

APEC pathotype can cause localized and systemic infections in poultry, but the pathophysiology of these diseases has remained unknown. Fimbrial and putative colonization factors in APECs is considered to be an essential step in their pathogenicity and is associated with resistance to heterophil cells activity.^5^

In this study, 8.82% of isolates were positive for *papE-F* gene. Stordeur *et al*. reported pap sequence in 91.30% of avian *E. coli* isolates.^6^ The *pap* genes have also been observed in a considerable frequency in APEC strains in the present and other studies.^11^^,^^12^ Some studies suggested an important role for P fimbria in pathogenicity of APECs.^5^ In Ireland, 41.20% of isolates from septicemic birds were positive for *pap* genes, compared to 15.60% from *E. coli *isolated from healthy birds.^13^ Pourbakhsh *et al*. showed the *in vivo *expression of P fimbriae in experimentally inoculated chickens and suggested that P fimbriae may be involved in colonization and development of septicemia.^14^

In the present study, five and two isolates were positive for *afaIB-C* and *sfa/focD-E* genes, respectively. Results of the present study is comparable with other reports, S and afa fimbriae coding genes have been detected less than 10.00% of APEC isolates.^4^^,^^15^ The S fimbria has been seldom founded in APECs and its pathogenesis is not exactly clear in poultry colibacillosis.^16^ A study showed a low prevalence of *afa *(5.50%) and *sfa *(4.40%) genes in APECs and suggested that the factors may have a considerable role in colonization.^6^ In Iran, Salehi and Ghanbarpour have reported the presence of virulence genes in fecal *E. coli* isolates from colisepticemic cases of Japanese quail and found the genes *afaI B-C*, *sfa/focD-E* and *papE-F* in one, four, and 10 isolates, respectively, which is similar to the results of the current study.^4^

Genotyping of APEC and evolutionary study of the adhesive capacity of *E. coli* strains isolated from avian colibacillosis indicated that these isolates can be classified into various phylogenetic groups.^4^ In the present study, the *E. coli* isolates mostly fell into the phylo-groups A followed by B1. Ewers *et al*. reported a higher amount (46.10%) of *E. coli* isolates from APEC that was belonged to A followed by B2 (35.10%) phylo-group.^15^ Another study on phylogenetic groups in *E. coli* strains isolated from septicemic broiler and layer cases indicated that the isolates were belonged to A (71.00%), B1 (4.10%), B2 (7.90%) and D (18.70%) phylo-groups.^17^ The phylogenetic background of 109 *E. coli* isolates from heart blood samples of dead quail has been evaluated by Salehi and Ghanbarpour in Iran in which 55.00% of isolates were belonged to A and the remaining were B1 (18.30%), B2 (17.40%) and D (9.20%) phylo-groups, that was similar to our results.^4^

According to the results of the present study, *E. coli* isolates from colibacillosis of Japanese quail were distributed in different phylogenetic groups, which contained few adhesins genes. However, avian colibacillosis might be associated with other virulence genes that was not examined. Further studies are needed to survey the phylogenetic background of *E. coli* isolates in comparison with their virulence genes to further refine the definition of pathogenic *E. coli*. Regarding limitation in collection of data in this species, findings of the present study could be helpful to understand the prevention and control of APEC in quail and to develop new and improved vaccines. 
